# Impact of litter size on sow stayability in Swedish commercial piglet producing herds

**DOI:** 10.1186/s13028-016-0213-8

**Published:** 2016-05-21

**Authors:** Emma Andersson, Jenny Frössling, Linda Engblom, Bo Algers, Stefan Gunnarsson

**Affiliations:** 1 Department of Animal Environment and Health, Swedish University of Agricultural Sciences, PO-Box 234, SE-532 23 Skara, Sweden; 2 Department of Disease Control and Epidemiology, National Veterinary Institute, SE-751 89 Uppsala, Sweden; 3 Department of Animal Breeding and Genetics, Swedish University of Agricultural Sciences, PO-Box 7023, SE-750 07 Uppsala, Sweden

**Keywords:** Productivity, Performance, Welfare, Health, Removal reason, Mortality

## Abstract

**Background:**

Sows’ ability to produce an excessive amount of piglets has shaped modern piglet production and there has been a steady increase in litter size during the last decades. This development has caused some negative side-effects, such as an increase in the proportion of stillborn piglets, a decrease in the proportion of weaned piglets and a larger variation in quality of piglets. Swedish commercial piglet producing herds have, like other countries with high production levels, high piglet mortality and high annual removal rate of gilts and sow. These problems seem to have increased during the same period that litter sizes have increased. Therefore present study aim to investigate whether there is an association between litter sizes and sow stayability.

**Results:**

The probability to produce ≥4 litters during a lifetime was significantly lower for sows giving birth to ≤8, 15 and ≥17 piglets in total in their first parity litter compared to sows giving birth to 13 piglets. Except for the group of sows having a small (≤11 piglets born in total) first parity litter size in combination with a medium (12–14 piglets born in total) second parity litter size, all other groups were significantly associated with an impaired ability to stay ≥4 litters compared to sows having a medium both first and second parity litter size. There were differences in removal reason between sows having small, medium or large first parities litter sizes.

**Conclusions:**

Associations between litter sizes in low parities and sow stayability were found. Our results indicate that aiming for keeping sows giving birth to a medium-sized litter, with approximately 12–14 piglets born in total may improve sows stayability and decrease the risk of unplanned removal. This should be considered when planning breeding strategy and annual removal in Swedish commercial piglets producing herds.

## Background

Sows’ ability to produce an excessive amount of piglets has shaped modern piglet production. The main breeding goal in the piglet production has, so far, been to increase the number of piglets born in each litter in order to improve the production efficiency. This strive has been successful as there has been a steady increase in litter size during the last decades [1]. However, this development has caused negative side-effects. Rutherford et al. [2] reviewed and listed welfare problems that concerns both piglets and sows due to large litter size. Problems include an increase in the proportion of stillborn piglets, a decrease in the proportion of weaned piglets and a larger variation in quality of piglets [2–4]. Effects of large litters on sows are more uncertain, but may include deterioration in the maternal ability [5] and impaired health and welfare of the sow [2].

Commercial piglet producing herds in Sweden as well as in many other countries have high production levels but also high piglet mortality and high annual removal rate of gilts and sows [1, 6]. The problems with high piglet mortality and sow removal rates seem to have increased during the same period that litter sizes have increased. The present study aims to investigate the association between litter sizes and sow stayability. Since the selection for increasing litter sizes mainly is based on the recordings on litter size in low parity numbers, the present paper will describe and evaluate the impact of first and second parity litter size on sow stayability and removal reasons.

## Methods

This study was performed as a retrospective study using data from a sow database established at the Swedish University of Agricultural Sciences (SLU). The database included production data from sows in Swedish commercial piglet producing herds. The herds participated in the data recording on a voluntary basis and exported their recorded production data from the herd monitoring program called PigWin Sugg (Quality Genetics HB, Hörby) to the SLU database once a year.

### Study population

Data from the database were extracted in January 2014. The data set was sorted by herd and sow identity and thereafter the quality of data was validated by use of descriptive statistics on dependent and independent variables included in this study. The source population consisted of 63,844 registered sows from 28 herds. In the database 71.2 % of the sows were crossbreed of Yorkshire and Landrace in different combinations, whereas 3.9 % were purebred of Yorkshire or Landrace and 24.9 % were crossbreed of Yorkshire, Landrace and Duroc or Hampshire, had missing data or had typing error. In total, five duplicates and 47 observations with biologically impossible typing errors (e.g., sows with more piglets born alive than born in total) were deleted. To be included in the study population sows had to be born between January 1, 1997 and December 31, 2009 (19,721 observations were deleted). This was done in order to analyse sows that potentially could produce at least 4 litters before the end of the study period (assuming sows being 1 year of age at first farrowing and giving birth to 2.2 litters a year [7]). Sows also had to be crossbreed (1 herd and 1545 observations were deleted) and had to produce at least one litter with the minimum of one piglet born in total (3089 observations were deleted). In order to be included in the dataset, individual herds had to contribute with ≥1 % of the observations (i.e., 3 herds compiling 559 observations were deleted). The final dataset included a study population of 38,878 sows in 24 herds. There were no data available of herd location, housing system or management, but according to the Swedish animal welfare legislation, crating is banned and sows must be loose housed during farrowing and lactation. The lactation period has to be at least 4 weeks. During the dry period, sows have to be loose housed in group pens. Furthermore, straw must be given daily to all pigs [6, 8].

For the whole study period (January 1997–January 2014), the median number of sows across herd was 1244 (range 577–5024 sows). The median number of sows across herd was 124 per year (range 1–836 sows). Twelve herds had records for the whole study period and the minimum number of years a herd had records was 8 years (Table 1). Among the herds, the median litter size, i.e., the total number of piglets born in a litter, varied between 12 and 13 piglets born in total for first parity sows and between 13 and 15 piglets born in total for second parity sows.

**Table 1:** Descriptive statistics of piglet production in 24 Swedish commercial herds

Herd	Year^a^	N sows	Breed^b^ (%)		Production performance, mean ± SD^c^				
			Unknown	Mix Y/L	Born in total	Stillborn (%)	Mortality (%)	Wean/sow/year	NPD
1	1997	1028	3.6	96.4	13.4 ± 2.3	6.7 ± 6.4	14.4 ± 23.7	22.7 ± 3.1	30.3 ± 28.1
2	1997	1132	22.4	77.6	12.9 ± 2.7	7.6 ± 8.6	8.5 ± 40.8	22.9 ± 4.0	43.6 ± 38.0
3	1997	938	68.6	31.5	12.8 ± 2.7	6.3 ± 7.2	12.1 ± 21.7	22.4 ± 3.6	25.7 ± 36.1
4	2006	984	0.0	100.0	13.5 ± 2.7	6.7 ± 7.4	10.3 ± 34.2	23.5 ± 3.8	28.6 ± 37.8
5	1998	1656	2.3	97.7	13.3 ± 2.4	5.4 ± 6.9	8.6 ± 32.9	24.1 ± 2.9	13.2 ± 27.3
6	2002	1175	0.6	99.4	13.7 ± 2.4	7.0 ± 7.1	15.0 ± 31.7	23.2 ± 5.0	35.8 ± 34.4
7	1997	764	100.0	0.0	13.2 ± 2.4	5.7 ± 6.9	13.0 ± 31.4	23.0 ± 4.5	40.6 ± 44.8
8	2004	881	0.2	99.8	14.3 ± 2.5	6.0 ± 6.0	13.5 ± 23.4	24.5 ± 2.4	19.0 ± 31.6
9	2006	1839	19.3	80.7	12.9 ± 2.8	8.8 ± 11.5	12.9 ± 38.7	21.3 ± 5.7	26.9 ± 34.2
10	1997	5024	0.4	99.6	12.5 ± 2.3	7.6 ± 9.2	7.7 ± 44.2	22.5 ± 5.2	25.1 ± 37.9
12	2000	577	6.2	93.8	13.0 ± 2.4	6.1 ± 7.4	11.0 ± 17.6	23.3 ± 3.5	27.7 ± 40.3
13	2003	1188	100.0	0.0	13.5 ± 2.7	8.5 ± 9.2	11.8 ± 31.3	22.7 ± 3.7	45.3 ± 52.6
14	2000	1457	34.9	65.1	13.5 ± 2.4	6.8 ± 6.3	8.1 ± 24.7	24.7 ± 3.7	24.7 ± 29.8
15	1997	1065	2.8	97.2	13.7 ± 2.5	6.2 ± 6.3	13.8 ± 23.4	23.4 ± 2.5	20.2 ± 25.8
18	1997	1299	16.0	84.0	12.8 ± 2.5	7.2 ± 8.5	11.7 ± 22.3	22.4 ± 3.7	21.9 ± 36.1
21	1998	2074	50.6	49.4	13.1 ± 2.5	5.6 ± 6.7	8.2 ± 30.4	23.8 ± 3.6	36.4 ± 50.5
22	2001	1124	3.2	96.8	13.5 ± 2.4	7.3 ± 8.7	15.7 ± 24.5	22.5 ± 4.3	27.4 ± 38.4
23	2001	2605	2.9	97.1	13.5 ± 2.6	6.9 ± 7.7	10.3 ± 39.6	23.7 ± 5.5	24.5 ± 33.9
24	1997	927	11.0	89.0	13.2 ± 2.3	7.1 ± 5.8	9.3 ± 23.2	23.8 ± 2.8	20.6 ± 34.4
25	1997	2288	77.3	22.7	13.4 ± 2.4	7.4 ± 6.3	13.1 ± 23.8	22.7 ± 3.0	37.7 ± 41.9
26	1997	1714	1.8	98.3	12.9 ± 2.4	4.0 ± 5.4	12.5 ± 21.1	23.0 ± 3.2	23.4 ± 37.2
27	2005	2232	9.8	90.2	14.1 ± 2.6	6.0 ± 7.0	12.7 ± 38.3	24.3 ± 5.1	29.2 ± 36.8
28	1997	1767	3.0	97.0	13.1 ± 2.6	6.0 ± 7.9	12.6 ± 28.4	22.8 ± 4.4	23.8 ± 31.8
29	1997	3140	11.1	88.9	13.2 ± 2.4	5.7 ± 8.0	12.6 ± 38.8	22.9 ± 5.1	25.0 ± 37.0

^a^Year when herd entered the study. Included sows were born between January 1 1997 and December 31 2009. All herds had records in 2013

^b^Percentage of breed in herd. Unknown = records having missing data, typing error or sow was crossbreed of Yorkshire, Landrace and Duroc or Hampshire, mix Y/L = crossbreed of Yorkshire and Landrace in different combinations

^c^Born in total = number of piglets born in total per litter, Stillborn = percentage of piglets stillborn per litter, Mortality = percentage of piglet mortality between birth and weaning, wean/sow/year = weaned piglets per sow and year (assuming sows giving birth to 2.2 litters a year [7]), NPD = total number of non-productive days in sows lifetime

### Litter size

As litter size differed between sows’ parities and selection for litter size mainly is based on records on litter size in low parity numbers, litter size in first and second parity litter was chosen as exposures of interest in the analyses. Mean litter size in first parity was 12.2 piglets (median 12, range 1–28) and in second parity mean litter size was 12.9 piglets (median 13, range 1–29). Over the study period median litter sizes increased in both first and second parity from 11 to 13 piglets and from 11 to 14 piglets, respectively, (Table 2).

**Table 2:** Number of piglets born in total in first and second parity litter size by birth year of sow

Birth year	Born in total, first parity			Born in total, second parity		
	N sows	Mean ± SD	Median (min–max)	N sows	Mean ± SD	Median (min–max)
1997	671	10.8 ± 2.4	11 (2–19)	646	11.2 ± 3.0	11 (2–20)
1998	1078	10.9 ± 2.6	11 (2–19)	1002	11.3 ± 3.1	12 (2–19)
1999	1233	11.4 ± 2.7	12 (1–23)	1085	11.5 ± 3.1	12 (2-28)
2000	1518	11.4 ± 3.0	12 (2–20)	1310	11.9 ± 3.3	12 (2–23)
2001	1887	11.5 ± 3.0	12 (1–23)	1630	11.9 ± 3.4	12 (1–24)
2002	3469	11.6 ± 3.0	12 (1–24)	2987	12.3 ± 3.4	13 (1–26)
2003	2689	12.1 ± 3.0	12 (1–28)	2234	12.8 ± 3.4	13 (2–25)
2004	3117	12.1 ± 2.8	12 (1–25)	2545	12.8 ± 3.3	13 (1–23)
2005	3266	12.1 ± 2.9	12 (1–23)	2796	13.1 ± 3.3	13 (1–23)
2006	5104	12.4 ± 3.0	13 (1–26)	4086	13.1 ± 3.5	13 (1–24)
2007	4688	12.7 ± 3.0	13 (1–24)	3877	13.5 ± 3.5	14 (1–29)
2008	4984	12.8 ± 3.1	13 (1–25)	4141	13.6 ± 3.6	14 (1–24)
2009	5174	13.0 ± 3.1	13 (1–25)	4371	13.9 ± 3.6	14 (1–27)
Total	38,878	12.5 ± 3.1	13 (1–28)	32,713	13.1 ± 3.6	13 (1–29)

Data selected from January 1 1997 to December 31 2009 from 24 Swedish piglet producing herds

In order to describe and evaluate the impact of litter size on sow stayability, first parity litter size was categorised into ten groups (≤8, 9, 10, 11, 12, 13, 14, 15, 16 and ≥17). The lowest and highest categories were based on the 10th and 90th percentiles of the distribution, respectively. The litter size born in total was categorised into small (S; ≤11 piglets), medium (M; 12–14 piglets) or large (L; ≥15 piglets) for analysis of the combined effect of the litter size in first and second parities. Based on these three categories, the litter size in first and second parity was combined into nine categories; small-small (S1S2), small-medium (S1M2), small-large (S1L2), medium-small (M1S2), medium-medium (M1M2), medium-large (M1L2), large-small (L1S2), large-medium (L1M2) and large-large (L1L2). In total, 32,713 sows out of all 38,878 sows in the study population had a second litter.

### Sow stayability

Stayability was defined based on the binary traits previously described by Serenius and Stalder [9]. In this study stayability was analysed as sows’ probability of producing a total number of litters in her lifetime higher or equal to the population median. A sows’ probability of having a second litter (considering her first parity litter size) or a third litter (considering the combined litter size based on first and second parity) was shown descriptively.

### Removal reasons

Removal date was recorded for 97.5 % of all sows during the study period. Herds could record 52 different removal reasons for sows. These removal reasons were grouped in the herd monitoring program to nine overall categories of removal reasons; sold/slaughtered, not pregnant, low yield, malformation/complicated farrowing, udder problems, bad temper, leg problem, traumatic injury and other reasons. The monitoring program enabled herds to record two reasons for removal of an individual sow. If a sow had two removal reasons registered, the first and main reason was included in the analyses. For sows recorded as “slaughtered” or “euthanized” as the first removal reason, the second reason, i.e., the primary reason why the sow was slaughtered or euthanized, was included in the analyses. For 1.4 % of the sows with removal date there was no removal reason recorded.

Firstly, sow removal was described regarding to whether the sow was euthanized or not. Secondly, removal reasons were described using nine categories of removal reason which previously has been analysed by Engblom et al. [6].

### Statistical analyses

The statistical software Stata (release 12, StataCorp LP, College Station, TX) was used both for data management and statistical analyses. The unit of interest was sow and litter size was the exposure of interest. In addition to descriptive statistical investigations, potential association between litter size and the probability of producing four or more litters in a lifetime was analysed using mixed-effects logistic regression.

Herd was included as a random variable in the models. Other variables that were considered to be of interest to control for in the primary models were birth year of the sow, age of first farrowing and season at first farrowing. Birth year of the sow and age of first farrowing, measured in days, were categorised according to quartiles; ≤2002, 2003–2006, 2007–2008, 2009 and ≤347, 348–363, 364–384, ≥385 days, respectively. There were 532 sows that had missing data regarding age at first farrowing. Season was categorised into winter, spring, summer and fall. Potential association between the outcome variable and these covariates were first assessed using univariable regression and then further investigated using multivariable regression. The final models were built using backward stepwise elimination. Variables with nonsignificant results (p > 0.05) were not included in the final models. Interaction between litter size and birth year of the sow was tested for in all the primary models, but was not significant and therefore not included in any of the final models.

## Results

### Effects of first parity litter size

#### Sow stayability

**Fig. 1:**
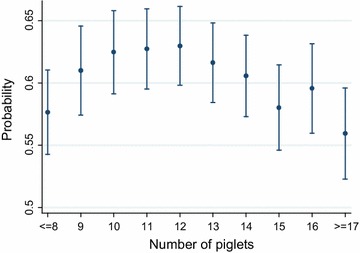
Predicted stayability and litter size in first parity. Predicted probability (with 95 % CI) of a sow producing ≥4 litters in her lifetime (*y-axis*) versus the total number of piglets born in her first parity litter (*x-axis*). Predictions are from multivariable logistic regression including number of piglets, birth year of the sow, age at first farrowing and season at first farrowing as explanatory variables and herd included as a random variable. Data were selected from January 1 1997 to December 31 2009 from 24 Swedish piglet producing herds and included 38,346 observations

Among sows giving birth to 9–16 piglets in their first parity, a higher proportion had a second litter and a higher proportion was able to stay ≥4 litters, compared to sows giving birth to ≤8 or ≥17 piglets (Table 3). The regression model of this outcome variable (i.e., sow stayability) showed significant negative associations between first parity litter sizes of ≤8, 15 and ≥17 piglets compared to sows giving birth to 13 piglets. Results from the regression model are shown in Table 4 and the predicted probability versus litter size is shown in Fig. 1.

**Table 3:** Descriptive statistics of sow stayability and removal reason according to first parity litter size

Number of piglets											
	Study population	≤8	9	10	11	12	13	14	15	16	≥17
Number of sows	38,878	4096	2244	3380	4520	5587	5514	4906	3747	2403	2481
Percentage of sows having ≥4 litters	59.6	56.4	60.2	61.5	61.8	62.1	60.7	59.7	57.2	58.6	54.5
Percentage of sows having a second litter	84.1	81.7	85.5	85.4	84.2	84.7	84.9	84.4	84.0	84.2	81.7
Number of sows	37,914	4024	2203	3326	4444	5447	5371	4760	3635	2313	2391
Percentage of sows being euthanized at farm	6.6	5.4	5.1	5.4	6.1	6.8	6.6	7.4	7.5	7.8	8.7
Reproductive disorders^a^	22.4	23.8	22.1	22.5	23.2	21.5	21.6	22.4	21.7	22.8	23.8
Low productivity^a^	7.9	11.2	8.3	7.2	6.9	7.7	7.3	7.4	7.6	8.0	7.8
Udder problems^a^	17.2	15.5	15.5	16.6	17.2	16.4	17.2	18.0	18.2	18.7	19.0
Lameness and/or foot lesions^a^	12.9	10.8	11.5	11.7	12.8	12.8	13.0	13.7	13.9	13.8	15.1
Traumatic injuries^a^	2.5	2.4	2.3	2.2	2.2	2.8	2.4	2.3	3.1	3.1	2.6
Inferior body condition^a^	2.3	2.5	2.7	2.0	2.3	2.5	2.5	1.9	2.5	2.3	2.4
Found dead^a^	4.3	4.6	4.5	4.0	4.0	4.6	4.5	4.6	4.0	3.9	3.8
Old age^a^	17.5	16.7	20.6	19.8	19.1	18.5	18.2	16.9	16.2	14.3	12.0
Miscellaneous^a^	13.0	12.5	12.5	14.0	12.3	13.2	13.3	12.8	12.8	13.1	13.5

Data selected from January 1 1997 to December 31 2009 from 24 Swedish piglet producing herds

^a^Removal category proposed by Engblom et al. [6]. Presented as percentage of sows

**Table 4:** Associations between first parity litter size and sows’ odds of producing ≥4 litters in her lifetime

Explanatory variable	OR	*P* value	95 % Conf. interval	
Categories				
Number of piglets				
≤8	0.81	0.000	0.74	0.88
9	0.92	0.118	0.83	1.02
10	0.99	0.834	0.91	1.08
11	1.01	0.760	0.93	1.10
12	1.05	0.266	0.97	1.13
13	Ref.			
14	0.97	0.509	0.90	1.05
15	0.89	0.008	0.82	0.97
16	0.96	0.443	0.87	1.06
≥17	0.85	0.001	0.77	0.94
Birth year of the sow				
≤2002	Ref.			
2003–2006	0.71	0.000	0.67	0.76
2007–2008	0.67	0.000	0.62	0.71
2009	0.68	0.000	0.63	0.73
Age (days) at first farrowing				
≤347	Ref.			
348–363	1.00	0.920	0.94	1.07
364–384	0.92	0.012	0.87	0.98
≥385	0.85	0.000	0.79	0.90
Season at first farrowing				
Winter	Ref.			
Spring	0.92	0.006	0.87	0.98
Summer	0.88	0.000	0.83	0.93
Fall	0.98	0.512	0.92	1.04

Estimates of odds ratio (OR) from multivariable logistic regression of a sow producing ≥4 litters in her lifetime. In addition to the explanatory variables listed in the table, herd was included as a random variable in the model. Data were selected from January 1 1997 to December 31 2009 from 24 Swedish piglet producing herds and included 38,346 observations

#### Removal reason

With an increasing litter size there was an increasing trend in proportion of sows being euthanized. Sows having ≤8 piglets was the largest group removed due to low productivity whereas sows having ≥14 piglets had the largest proportions of sows removed due to udder problems. Problems with lameness and/or foot lesions increased in proportion with increasing litter size. It was found that 12.0 % of sows giving birth to ≥17 piglets were removed due to old age compared to 20.6 % of sows giving birth to nine piglets in their first parity litter (Table 3).

### Combined effect of first and second parity litter size

#### Sow stayability

**Fig. 2:**
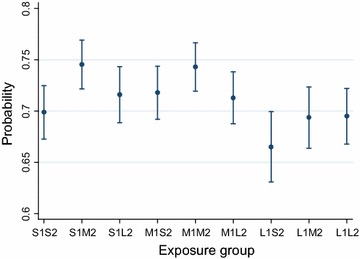
Predicted stayability and litter size in first and second parities. Predicted probability (with 95 % CI) of a sow producing ≥4 litters in her lifetime (*y-axis*) for different exposure groups based on combined categories of first and second parity litter size (*x-axis*): *S* small litter size (≤11 piglets), *M* medium litter size (12–14 piglets), *L* large litter size (≥15 piglets), *1* first parity and *2* second parity. Predictions are from multivariable logistic regression including exposure group, birth year of the sow and age at first farrowing as explanatory variables and herd as a random variable. Data were selected from January 1 1997 to December 31 2009 from 24 Swedish piglet producing herds and included 32,300 observations

Group S1M2 and M1M2 had a higher proportion of sows having a third litter and a higher proportion of sows that was able to produce ≥4 litters than the other groups, see Table 5. With the exception of sows in group S1M2, all groups were significantly associated with an impaired ability to produce ≥4 litters compared to sows in the M1M2 group. Results from the regression model are shown in Table 6 and the predicted stayability versus litter size in first and second parity litter is shown in Fig. 2.

**Table 5:** Descriptive statistics of sow stayability and removal reason according to first and second parity litter size

	Study population	Exposure group^b^								
		S1S2	S1M2	S1L2	M1S2	M1M2	M1L2	L1S2	L1M2	L1L2
Number of sows	32,708	4633	4526	2800	3870	5024	4661	1392	2186	3616
Percentage of sows having ≥ 4 litters	70.9	69.2	74.0	70.7	71.1	73.8	70.6	65.7	68.5	68.7
Percentage of sows having a third litter	85.6	84.1	87.5	85.6	85.3	86.7	85.9	82.4	85.0	85.3
Number of sows	31,748	4586	4427	2703	3777	4870	4479	1358	2110	3438
Percentage of sows being euthanized at farm	5.5	4.1	4.5	6.0	4.7	5.7	6.4	6.3	6.2	6.9
Reproductive disorders^a^	19.3	19.7	20.5	18.4	20.4	17.9	18.8	21.2	19.7	18.7
Low productivity^a^	9.1	12.6	7.3	8.2	9.1	8.2	8.9	9.3	8.4	9.7
Udder problems^a^	18.6	16.8	18.2	18.4	18.5	18.1	19.4	20.7	17.8	21.4
Lameness and/or foot lesions^a^	11.5	9.9	10.6	12.0	10.4	11.9	12.6	9.9	13.1	13.0
Traumatic injuries^a^	2.0	1.8	1.9	2.1	2.0	1.8	2.0	2.1	2.2	2.3
Inferior body condition^a^	2.2	2.0	1.9	2.4	2.2	2.4	2.0	3.2	1.9	2.2
Found dead^a^	3.8	3.4	3.8	4.1	3.8	4.3	3.8	3.2	3.9	3.5
Old age^a^	20.9	22.0	23.9	20.9	21.5	22.7	19.4	15.8	20.7	16.1
Miscellaneous^a^	12.6	11.8	11.9	13.5	12.1	12.7	13.1	14.6	12.3	13.1

Data selected from January 1 1997 to December 31 2009 from 24 Swedish piglet producing herds

^a^Removal category proposed by Engblom et al. [6]. Presented as percentage of sows

^b^S = small litter size (≤11 piglets), M = medium litter size (12–14 piglets), L = large litter size (≥15 piglets), 1 = first parity and 2 = second parity

**Table 6:** Associations between first and second parity litter size and sows’ odds of producing ≥4 litters in her lifetime

Explanatory variable	OR	*P* value	95 % Conf. interval	
Categories				
Exposure group^a^				
S1S2	0.75	0.000	0.68	0.82
S1M2	0.98	0.683	0.89	1.08
S1L2	0.89	0.025	0.80	0.99
M1S2	0.86	0.002	0.78	0.94
M1M2	Ref.			
M1L2	0.89	0.012	0.81	0.98
L1S2	0.71	0.000	0.62	0.81
L1M2	0.82	0.000	0.73	0.91
L1L2	0.84	0.001	0.77	0.93
Birth year of the sow				
≤2002	Ref.			
2003–2006	0.78	0.000	0.73	0.83
2007–2008	0.69	0.000	0.64	0.75
2009	0.68	0.000	0.62	0.74
Age (days) at first farrowing				
≤347	Ref.			
348–363	1.00	1.000	0.93	1.07
364–384	0.95	0.141	0.88	1.02
≥385	0.87	0.000	0.81	0.94

Estimates of odds ratio (OR) from multivariable logistic regression of a sow producing ≥4 litters in her lifetime. In addition to the explanatory variables listed in the table, herd was included as a random variable in the model. Data were selected from January 1 1997 to December 31 2009 from 24 Swedish piglet producing herds and included 32,300 observations

^a^S = small litter size (≤11 piglets), M = medium litter size (12–14 piglets), L = large litter size (≥ 15 piglets), 1 = first parity and 2 = second parity

#### Removal reason

The proportion of sows being euthanized was higher in the groups having a large first or second parity litter than in the other groups (6.0–6.9 and 4.1–5.7 %, respectively). Sows having a large litter in first or second parity also had the lowest proportion of sows being removed due to old age (Table 5). The proportion of sows being removed due to lameness and/or foot lesions increased with an increasing second litter size, see Table 5.

## Discussion

The aim of this study was to investigate the association between litter size in the first parities and sow stayability. The impact of first parity litter size on sow stayability and removal reasons was evaluated. A negative association between litter size and stayability was found amongst sows giving birth to a large or small number of piglets in any of their first two parities, except for sows having a small first parity litter size in combination with a medium second parity litter size. Furthermore, it was found that sows having large litter sizes in their first two parities were more often removed due to unplanned reasons and that sows with small litter sizes were more likely to be removed due to planned reasons. Our results imply that larger litters are not necessarily better than medium sized litters. The results from this study suggest that Swedish pig producers would benefit from aiming for keeping sows giving birth to a medium-sized litter, with approximately 12–14 piglets born in total, as this seems to improve their stayability and also decrease the risk of unplanned removal. This should be considered in the planning of breeding strategies and annual removal of sows.

By using a database comprising records from 28 commercial piglet producing herds in Sweden and 15 years of data a large study sample was achieved. To use an already established database has advantages such as being readily available and saving time and money. However, there are also disadvantages that need to be considered; e.g., data was not recorded for our specific research questions and the recording was beyond our control. All participating herds are kept anonymous in the sow database used in this study. Therefore, no data of herd location, housing system and management was available or possible to retrieve retrospectively. However, it can be assumed that the sows were kept according to Swedish legislation, i.e., in loose housing systems both during gestation, farrowing, lactation and non-productive days, and that the lactation period was at least 4 weeks long. Due to the fact that the recording was made by different persons, the robustness of data could be expected to be moderate and therefore, the indicators of our interest were selected based on their relevance, completeness and consistency.

In this study the sows were crossbreed in various combinations. About one quarter of the observations in the source population had missing information about breed and was mainly associated with specific herds. We assume that these sows mainly were crossbreed of Yorkshire and Landrace in various combinations or crossbreed of Yorkshire, Landrace and Duroc or Hampshire and chose to include all of these sows in the study since it reflected the typical Swedish commercial piglet production. However, purebred Yorkshire or Landrace were excluded from analysis as these breeding herds often have different removal strategies, as their production aims are different from herds producing piglets for slaughter. We postulate that our results of associations may be applicable on most pig breeds but our categorisation of a small, intermediate and large litter size probably applies mainly on crossbreed Yorkshire and Landrace sows that are held under similar extensive production as Swedish commercial piglet production. In order to define if the same association exists in other breeds needs to be further investigated.

Litter size in first and second parity was chosen to be the exposure in the analyses. In general, unplanned removals of sows are performed before the sows have produced their third litter [6]. Therefore, the first and the second litters were considered the most interesting litters to study from a welfare and health perspective. Furthermore, other studies show that sow performance based on the first litter provide insight into the rest of the sow’s productive life [10]. In addition, sows with large first parities litter size have been shown to continue to have large litter sizes during their lifetime [11]. In the companion reviews of Rutherford et al. [2] and Baxter et al. [12] it was concluded that when assessing the effects of litter size on sow welfare, it is important to consider both the number of piglets born alive as well as stillborn piglets, because it wears the sow carrying and giving birth to the large litter. Rutherford et al. [2] and Baxter et al. [12] classified 7–13 piglets to be a small/medium sized litter and 14 piglets or more as large or very large litter sizes. These authors also argue that the average number of 14 functional teats seen in current sows should be the upper limit of a litter size and this statement is also supported by Chalkias et al. [13]. The Animal Health and Welfare panel of the European Food Safety Authority concludes that large litters pose a major welfare problem both for the piglets and for the sows, and the panel recommendation for genetic selection is that a litter should not exceed 12 piglets born alive on average (approximately 13 piglets born in total counting with less than 10 % piglets being born dead) [14]. Furthermore, Andersen et al. in [15] suggest that 10–11 piglets is the maximum of what a domestic sow may be capable of taking care of during the lactation period. Aiming for an average of less than 6 % stillborn piglets and less than 14 % piglets dying between birth and weaning, gives a maximum of approximately 13 piglets born in total. This study supports the idea that there is a maximum to the number of piglets a sow should give birth to in order to be sustainable, and that this maximum is around 12–14 piglets.

Previously it has been found that sows that stay in the herd for a longer period have a prolonged productive lifetime and are more profitable for producers than sows with a shorter productive lifetime [16, 17]. Results from our regression models suggest that a first parity litter size of 9–14 piglets born in total increases sows’ stayability. In a questionnaire study from 2014 Swedish commercial piglet producers were asked how they experienced their profitability [18], and it was found that the producers that answered that they had experienced good profitability weaned on average 24.3 piglets per sow and year. This corresponds to approximately 11 weaned piglets in a litter, given that sows in Sweden produces approximately 2.2 litters every year [7]. Producers reporting that they experienced poor profitability weaned on average 10.5 piglets in a litter. Therefore, aiming for a minimum of 12 piglets born in total in a litter may be considered a relevant reference for herd profitability and the lower limit of a moderate litter size, instead of a minimum of nine piglets born in total. Aiming for 12–14 piglets as a medium litter size seems relevant from a productivity and a stayability perspective.

A large proportion of the sows in our study were removed already after their first or second litter. Based on the fact that sows have to produce at least 3 litters before they provide a positive income for the producer [16], our results indicate that, based on first parity, a very small or a very large litter size have a negative effect on sow productive lifetime and these sows are non-profitable. Furthermore, sows that had a small second litter size and sows that had a large first and/or second litter size had an impaired stayability compared to sows that had medium litter sizes. Sows with a small second litter were more often removed due to low productivity and/or old age, i.e., causes that can be categorised as planned removal by the farmer. Sows with large first and second litter sizes were more likely to be removed due to unplanned reasons such as udder problems, lameness and/or foot lesions. These findings are supported by research of Engblom et al. [6] that also concludes that planned removals are less likely to be linked to impaired health and welfare compared to unplanned reasons. It has also been shown that improved pig health in piglet producing herds has a positive effect on the average number of litters born, the number of stillborn piglets and the number of weaned piglets [19]. Together with our results this indicates that there may also be an association between litter size and sow health and welfare, which needs to be further investigated.

## Conclusions

Associations between litter sizes in low parities and sow stayability was found. Our results indicate that aiming for keeping sows giving birth to a medium-sized litter, with approximately 12-14 piglets born in total may improve sows stayability and decrease the risk of unplanned removal; and this should be considered when planning breeding strategy and annual removal in Swedish commercial piglets producing herds.
